# Fogging to combat dengue: factors influencing stakeholders' attitudes in Malaysia

**DOI:** 10.1186/s12889-023-16054-3

**Published:** 2023-06-14

**Authors:** Ahmad Firdhaus Arham, Latifah Amin, Muhammad Adzran Che Mustapa, Zurina Mahadi, Mashitoh Yaacob, Mohamad Muhidin Patahol Wasli, Noor Sharizad Rusly

**Affiliations:** 1 Pusat Pengajian Citra Universiti, Universiti Kebangsaan Malaysia (UKM), Bangi UKM Bangi, Selangor, Malaysia; 2 The Institute of Islam Hadhari (HADHARI), Universiti Kebangsaan Malaysia (UKM), UKM Bangi, Selangor, Malaysia; 3 Centre for Agro-Food Economy and Development (CREDA), Universitat Politècnica de Catalunya (UPC), Casteldefells, Spain; 4 UCL Institute of Education, University College London, London, UK

**Keywords:** Attitude, Stakeholder analysis, Fogging technique, Dengue disease, PLS-SEM, Malaysia

## Abstract

**Background:**

Fogging is a conventional technique used to combat Aedes mosquitoes and prevent dengue disease. It is often implemented in outbreak areas or areas harbouring a high density of Aedes mosquitoes. Currently, studies on stakeholders' attitudes towards fogging are still limited in number. Therefore, this study aims to assess Malaysian attitudes, and identify the predicting factors influencing such attitudes.

**Methods:**

A validated instrument was used to interview 399 randomly selected respondents from the public (*n* = 202, 50.6%) and scientists (*n* = 197, 49.4%) in the Klang Valley region of Malaysia. The data were analysed using PLS-SEM involving Smart-PLS software.

**Results:**

The results confirmed that stakeholder attitudes toward fogging should be viewed in terms of a multi-dimensional association. The stakeholders surveyed were highly positive with regard to the application of fogging to control dengue but professed moderate concerns as to associated risks. The PLS-SEM analyses demonstrated that the perceived benefit was the most important factor influencing attitudes, followed by trust in key players.

**Conclusions:**

This result provides a good insight from the perspective of education and unravels the underlying fundamentals of stakeholders' attitudes toward the fogging technique. The findings also provide a positive indicator to the responsible parties involved to continue the usage of this technique in conjunction with improvements with regard to its safety aspects, and possibly in combination with other environmental-friendly alternatives in order to achieve a healthy environment without dengue in Malaysia.

**Supplementary Information:**

The online version contains supplementary material available at 10.1186/s12889-023-16054-3.

## Introduction

Dengue is the main vector-borne disease affecting tropical and subtropical countries worldwide, including Malaysia [1, 2]. Globally, dengue incidences have increased to 100 million cases, with up to 500,000 cases of haemorrhagic dengue recorded each year [3, 4]. In Malaysia in 2019, the number of dengue cases increased to a total of 130,101 (with 182 deaths) as compared to the previous year during which 80,615 cases (with 147 deaths) were recorded [5]. However, in Malaysia, dengue cases began to decline in 2020, with a total of 90,304 dengue cases with 145 deaths. The number of cases decreased even further in 2021, with a total of 21,455 cases reported up to 31 October 2021 [5]. This may be due to the Movement Control Order (MCO) enacted by the Malaysian government to control the spread of the COVID-19 pandemic that affected many countries worldwide. The closure of various sectors caused Malaysians to stay at home, resulting in a declining number of dengue cases. However, there are concerns that the number of such cases will have increased now that Malaysians have been given the flexibility to work and socialise due to the declining COVID-19 cases and increasing number of vaccinations. However, we do not want to return to the situation in which dengue cases were rampant, such as between 2014 and 2019. Currently, there is no effective vaccination against dengue fever or new methods for controlling the Aedes mosquito population. Having said this, to date, aside from the fogging technique, several methods have been practised in Malaysia for the mitigation of dengue diseases. These include the use of Abate larvicide, Precision Time Protocol (PTP)/source reduction, Temephos EC spray, Outdoor Residual Spray (ORS) for hotspot areas, and the enforcement of the Destruction of Disease–Bearing Insects Act (DDBIA) [6]. Fogging is carried out based on reported viral cases [1, 7]. The mosquito fogging service in Malaysia is undertaken by qualified practitioners from the Ministry of Health, staff from the local municipality, or by Pest Control Operators (PCO) from private companies. To date, there are many such companies in Malaysia offering mosquito fogging services, such as Empire Pest Control, Rapidkill, Kamal & Kamal Pest Control, and Pesco Pest Control.

There are two types of fogging techniques: basic thermal spray (thermal foggers) and Ultra Low Volume (ULV) spray (cold foggers). The thermal spray technique interferes with the viral transmission cycle by killing its main vector within 24 h. This technique should be initiated immediately the first case is reported. The fogging disinfectant is sprayed on indoor and outdoor premises that are potential breeding sites of the Aedes mosquitoe [2]. ULV foggers convert the fogging liquid to a fine mist which is dispersed into the air at high velocity. The advantage of cold foggers are their ability to fog both indoors and outdoors safely, as there is reduced risk of asphyxiation and it tends to be more effective than other methods [8, 9]. On the other hand, ULV spray-based fogging is performed on a large-scale, extending across a wide range of areas, with longer-lasting effects.

Many studies have been performed in Malaysia to assess various aspects of knowledge, attitudes, and practices (KAP) with regard to dengue prevention [10–16]. For instance, Al-Dubai et al. [10], Al-Hoot et al. [12], and Kamel et al. [13] noted that respondents believed that the fogging technique performed by the municipal council was not efficient when it came to preventing dengue because they were not convinced of the effectiveness of this technique. Wong et al. [11] indicated that respondents agreed that infrequent fogging allowed an increase in dengue cases. On the other hand, 58.7% of respondents did not believe that fogging was the only method available with regard to controlling dengue [14]. In contrast, Amin et al. [17] indicated that stakeholders in the Klang Valley of Malaysia displayed a positive attitude towards fogging. They indicated that this technique was beneficial, and trusted the key players involved in using this technique to control dengue. However, they noted that this technique posed a moderate risk. In another study by Selvarajoo et al. [16], a total of 89.7% of the respondents showed interest in reducing dengue cases. However, 49.6% of the respondents agreed that fogging was adequate in preventing dengue infection while the remaining 50.4% disagreed and were unsure if the technique was good enough. In terms of practice, 92.5% of the respondents agreed to contact the authorities to carry out fogging to control the transmission of dengue. Similarly, Arham et al. [18] indicated that 89.7% of respondents in the Klang Valley answered 'yes' when it came to expressing their support for the scheduled fogging technique. However, they seemed to have a moderate level of engagement through knowledge, awareness, and past intended behaviour relating to dengue control techniques. This study proposed that good stakeholder participation promotes good governance on the part of the municipal authority when it came to making use of dengue control techniques.

There has been a great deal of debate on how to define stakeholders. According to Burton [19], stakeholders are groups who have expert knowledge that should be taken into account, and are crucial to the execution of the resulting policies, and/or have a stake in the project's success. Other definitions emphasise the stakeholders’ ability to affect an organisation, project, or policy direction [20]. Meanwhile, in the context of comparative effectiveness research, Deverka et al. [21] offered a definition of stakeholders as "individuals, organisations or communities that have a direct interest in the process and outcomes of a project, research or policy endeavour". Therefore, in this study, we define stakeholders as individuals who affect successful fogging techniques, in the form of two groups: the general public and scientists.

The findings of previous studies indicate that Malaysians have an excellent attitude with regard to dengue prevention. They also have a high trust in the authorities when it comes to performing fogging, but they remain unsure of the effectiveness of this technique. The findings have also shown different perspectives among respondents in terms of demographic factors and times. Therefore, the use of structured equation modelling on attitudes towards the fogging technique using Smart-PLS software is very timely as a means of investigating the association between the predictor factors that influence stakeholders' attitude towards the fogging technique.

### The conceptual framework

The conceptual framework of the study was based on Amin and Hashim's [22] model of stakeholders' attitudes regarding genetically modified Aedes mosquitoes, which in turn was based on Fishbein's Multi-Attribute Attitude Model [23]. The model was created by compiling a list of the predictor elements that influence opinions regarding the fogging technique. In this model, the perceived benefits and risks were the specific predictors, while the attitudes toward nature versus material, trust in key players, and religiosity were the general predictors [22, 24–27]. The component-based approach of the PLS-SEM involving Smart-PLS software was performed to determine associations among the predictors.

### Hypotheses development

**Fig. 1:**
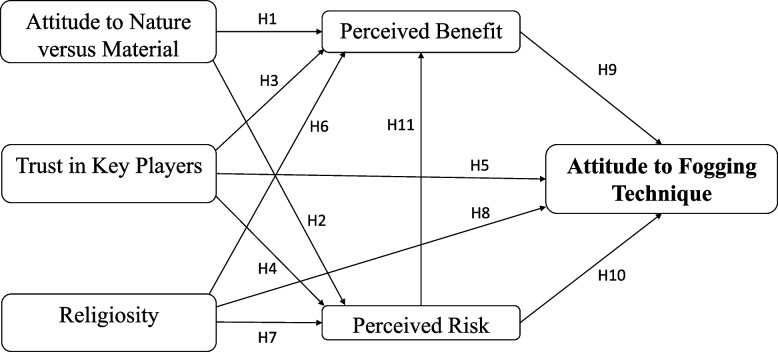
The conceptual framework

The research hypotheses of the conceptual framework as shown in Fig. 1 were developed to examine the links between the predictor factors using the Pearson's correlation approach [28]. In order to investigate the associations, each construct with regard to the predictors consisted of multiple items in the research model.

To evaluate the stakeholders' acceptance of the fogging technique, five items were listed to evaluate their attitudes (α = 0.886); a) fogging helps to decrease fatality in the community; b) fogging is necessary; c) fogging is encouraged; d) fogging activities should be increased; and e) the government should provide more financial support to researchers and industry to develop the fogging technique. All items were evaluated on a seven-point Likert scale, ranging from 1 (strongly disagree) to 7 (strongly agree).

### General factors

The first factor evaluating the stakeholders' attitudes toward nature *versus* material (α = 0.857) is comprised of five items: a) a society prefers to preserve nature or use nature to achieve wealth; b) a society with a centrally-planned economy or a market-driven economy; c) a society that will stop development at the expense of any risks or accepting any risks in the attainment of wealth; d) a society that optimises the protection of the environment above the economic growth or otherwise; and e) a society that understands that nature is fragile and can be easily damaged by human actions or can withstand human actions. All items were graded on a seven-point Likert scale ranging from 1 (preferring nature) to 7 (preferring technology). A higher grade indicated that people were more materialistic rather than being concerned with nature preservation. The attitude towards nature refers to the respondents' inclination to either maintain nature or to emphasise materialistic gains, be less concerned about the environment, and support modern technology [25, 29]. This factor related to the support of societal values or nature values [25, 30]. Previous studies have shown that more materialistic people tend to support the benefits of modern technology in terms of dengue control techniques, even though they are perceived as being risky [17, 22, 31]. As a result of this factor's importance based on the correlation results, the following theories were proposed:

- *H1: Malaysians who tend to be more materialistic will perceive more benefits from the fogging technique.*
- *H2:Malaysians who tend to be more materialistic will perceive fewer risks associated with the fogging technique.*

The second factor, trust in key players (α = 0.791), is comprised of three items: a) scientists and researchers have achieved a good outcome for society; b) pesticides and pharmaceutical industries have achieved a good outcome for society; c) government sectors involved in introducing relevant regulations such as the Ministry of Health and the Biosafety Department have achieved a good outcome for society. All items were scored on a seven-point Likert scale ranging from 1 (very low trust) to 7 (extremely high trust). Trust in experts is a common factor for technology acceptance [32]. Industry experts play a role in delivering information to the public by directly assessing the benefits and risks of a particular technology [33]. Earle & Cvetkovich [34] reported that consumers managed risks related to a technology based on their trust in experts and institutions related to the technology. Amin et al. [35] and Arham et al. [18] also showed that when people had more trust in key players, they would perceive more benefits associated with the technologies. Amin and Hashim [22] indicated that trust in key players had prompted the stakeholders included in their research to regard the genetically modified Aedes mosquito technique as beneficial. As a result, they demonstrated a positive attitude towards this technique. As a result of this factor's importance based on the correlation results, the following theories were proposed:

- *H3:Malaysians who have more trust in key players will perceive more benefits from the fogging technique.*
- *H4:Malaysians who have more trust in key players will perceive fewer risks associated with the fogging technique.*
- *H5:Malaysians who have more trust in key players will have a more positive attitude towards the fogging technique.*

Religiosity (α = 0.947), on the other hand, is comprised of eight items: a) religion is important in my life; b) religious views are important when I have to make decisions about controversial issues; c) praying is important in my life; d) reading scripture is important in my life; e) religion is important to answer any questions about the meaning of life; f) religion offers comfort when sorrow and misfortune strike; g) I try hard to live all my life according to my religious beliefs; h) nothing can occur without God's involvement in the process. All items were measured on a seven-point Likert scale ranging from 1 (lower level of religiosity) to 7 (greater level of religiosity). Religiosity is essential in shaping people's ethics and life [36, 37]. Tiliouine et al. [38] stated that religiosity could provide positivity and satisfaction to a person. Religiosity refers to a ritualistic orientation and to doctrinal influences in decision-making [35], demonstrating the importance of religiosity in determining an individual's consent to a particular matter. Psychologists claim that religiosity factors can shape the perception and attitudes of people regarding the world [39], thus helping them resolve uncertainties in such a way as to maintain the well-being of the community [40]. The stakeholders in the Klang Valley showed a high degree of commitment to their religion (with a mean score 6.07) in terms of the evaluation of their attitude towards fogging. However, Amin and Hashim [22] found no relationship between religiosity factors and stakeholders' attitudes toward the genetically modified Aedes mosquito. On the other hand, Amin et al. [29] noted a positive correlation between religiosity and perceived risk, thereby indicating that respondents who had more religious affiliations would perceive increased risk with regard to any new technologies. At the same time, Arham et al. [31] discovered that religiosity plays a mediating role in terms of the effect of perceived benefit and risk on stakeholders' attitudes toward the ORS technique for dengue control. Therefore, due to the importance of this factor based on the correlation results, the following hypotheses were proposed:

- *H6:Malaysians who are more religious will perceive more benefits from the fogging technique.*
- *H7:Malaysians who are more religious will perceive fewer risks associated with the fogging technique.*
- *H8:Malaysians who are more religious will show a more positive attitude towards the fogging technique.*

### Specific factors

In this model, the stakeholders' attitudes toward fogging were determined by the specific perceptions of benefit and risk. Rowe [41] noted that perceived benefits and risks were the major contributing factors in determining public attitude. Benefits are often related to the profits gained by society, producers, or consumers, but the risks are associated with the ethical issues that disrupt the environment or adversely affect health [35]. Fischhoff et al. [42] indicated that the perceived benefits and risks have consistent relationships that are difficult to conceptualise separately. Alhakimi and Slovic [43] reported an inverse relationship between perceived benefit and risk. In addition, Amin and Hashim [22], Amin et al. [17], and Arham et al. [31] showed that the perceived benefit was regarded as a significant factor in terms of the public acceptance of dengue prevention techniques.

Seven items were used to measure perceived benefit (α = 0.886): a) fogging will enhance the quality of life; b) fogging is useful to Malaysian society; c) fogging is useful in preventing dengue fever; d) fogging is effective in eradicating dengue; e) fogging is beneficial to me and my family's health; f) the benefits of fogging far out-weigh the risks; and g) the risks associated with fogging will be addressed by future research studies. All items were scored on a seven-point Likert scale, with 1 being the least beneficial and 7 being the most beneficial. The public is likely to be supportive of various technologies depending on the perceived benefits. Gaskell et al. [25, 26] found that respondents were receptive with regard to biotechnology applications based on the perceived benefits. Due to the importance of the perceived benefits of fogging, the following hypothesis was proposed:

- *H9:Malaysians who see more benefits will have a more positive attitude towards the fogging technique.*

The perceived risk (α = 0.874) was measured in terms of seven items: a) level of uncertainties regarding the unknown effects of fogging; b) any harmful effects from fogging will only manifest themselves in the long term; c) fogging will pose threats to future generations; d) fogging may have unknown consequences; e) any danger from fogging may cause a major catastrophe to Malaysian society; f) the extent of concern a person has about the potential risks of fogging to their health; and g) the adverse effects of fogging are harmful. All items were scored on a seven-point Likert scale, with 1 representing minimum risk and 7 representing highest risk. Perceived risk is a negative predictor of attitude, in that it refers to the loss that an individual will suffer from as the result of an unfavourable activity or event [44]. Slovic et al. [45] described the characteristics of perceived risk as involving aspects that cause fear, lack of precaution, death or disaster, threats to future generations, and various other risks. Therefore, the following hypotheses were proposed:

- *H10:Malaysians who perceive more risks will have a more negative attitude toward the fogging technique.*
- *H11:Malaysians who perceive more risks will perceive fewer benefits with regard to the fogging technique.*

### Research methods

From September 2016 to September 2017, respondents from the public and scientists aged 18 and above participated in a questionnaire-based survey. Combining these two stakeholders are important as they have similar interests as major potential beneficiaries of the fogging technique. Respondents were chosen using a purposive sampling method so that the sample could give important information while minimizing sampling error [46]. G*Power software was used to determine the sample size [47]. The results suggested that this study only required a minimum sample size of 277 respondents based on a statistical power of 0.80, effect size (f = 0.15), and significance levels (*p* < 0.05) [48]. Since the required sample is 277 and requires samples from the public and scientists, the ideal number required is 139 samples for each sample category. This study focused on the total number of respondents as 50% of the sample consisted of the public, and 50% of the respondents targeted scientists according to the maximum sample size required.

The Klang Valley was selected as the study's location because it has the highest number of reported dengue cases in Malaysia, according to the Malaysian Ministry of Health (http://idengue.arsm.gov.my). Therefore, individuals from the Klang Valley community who lived in outbreak areas or areas with high Aedes mosquito densities were selected as respondents. However, the location listing according to the six targeted areas (Petaling, Hulu Langat, Gombak, Klang, Sepang, the federal territory of Kuala Lumpur, and Putrajaya) that have the highest dengue hotspot localities in the Klang Valley are listed first to avoid bias in the selection of the public and scientists.

The public was chosen purposively based on the odd-numbered place of residence on the prepared list. After entering the residential area that has been selected for sampling, the researcher chooses a house in the middle of the row of land houses or the middle of the level of multi-story houses. A total of 40 samples of the public were targeted to be respondents from the six targeted areas of the study location. The scientists involved in this study consist of academics and researchers in district offices, hospitals, and universities in the Klang Valley in the fields of environmental science, biological science, and health science, as well as those involved in dengue control because they are directly and indirectly involved in dengue control and prevention activities. To reduce bias, listing the target location of the study to obtain a sample of scientists from six targeted areas in the Klang Valley is highly emphasized. This study decided to get purposively respondents of 40 samples of scientists from the list that has been prepared according to the six locations that have been targeted. However, the scientists' response depends on their agreement to receive the questionnaire to answer.

Prior to participating in the actual study, all respondents gave informed consent, verbally and voluntarily. Meanwhile, to ensure all the questions were addressed correctly, the respondents filled out this questionnaire with the help of the researchers and enumerators. The participation of the respondents was voluntary, and they were allowed to withdraw at any time. There were no inclusion or exclusion criteria used in this study, this is because any respondent among the public and scientists who are in the six locations that have been set has the opportunity to participate in this study as a respondent. A total of 415 questionnaires were successfully returned, of which 213 were responses from the public and 202 questionnaires were responses from scientists. However, only 399 sample respondents were used after discarding some doubtful responses and leading to external element data that could affect the results of the study. Therefore, the final sample size of 399 used in this study involving the public (*n* = 197) and scientists (*n* = 202) considered sufficient.

The research instruments used in this study were tested in two phases. First, seven experts in environmental management, sustainability governance, environmental health and science, and consumer behaviour related to dengue control and prevention, evaluated the validity of the survey content. They were given instructions as to how to assess the appropriateness, relevance, and representativeness of the survey items to ensure validity. The survey's two-way translation (English-Malay version) was then verified by two language experts. Next, a pilot test was performed with a total of 126 respondents, and some changes were made to the measurement items based on their comments and feedback. The finalized instrument items are as shown in Additional file 1.

The multi-dimensional instrument used in this study to measure the attitude towards fogging was constructed based on previous studies [22, 29, 49]. The instrument was validated by seven experts in various fields such as environmental studies, health sciences, bioethics, and society. All the items were measured on a 7-point Likert scale. After the completion of the data collection, the data management phase involved the use of SPSS version 24 to produce descriptive statistical analyses. Data analysis was performed by measuring the instrument items to confirm the reliability and validity of the research constructs, and to analyse the associations between the predictors in the proposed research model using the Smart-PLS software.

## Results

The mean score for each factor was examined prior to exploring the relationship between the factors (Table 1). In this study, the stakeholders in the Klang Valley region showed a higher mean score for three predicting factors. The religiosity factor showed the highest mean score (6.07), thus indicating that respondents had a high affiliation to their religion. They also showed a high level of trust in key players with a mean score of 5.51, such as the government, research institutions, and pesticide and pharmaceutical companies involved in controlling dengue. The perceived benefit of fogging was also categorised as being high (mean score of 5.37). However, they also acknowledged that fogging posed moderate risks (mean score of 3.18). Additionally, the Malaysian stakeholders professed that they were more inclined towards nature (mean score of 3.91). Overall, the stakeholders expressed their tremendous support for fogging as indicated by the high mean score of attitudes toward the fogging technique.

**Table 1:** Mean score of the predictor factors and attitude to fogging technique

Factors	Mean Score ± Standard Deviation	Interpretation
Attitude to fogging technique	5.38 ± 1.05	High
Perceived benefit	5.37 ± 1.16	High
Perceived risk	3.18 ± 1.36	Moderate
Trust in key players	5.51 ± 0.94	High
Attitude to nature versus material	3.91 ± 1.42	Moderate
Religiosity	6.07 ± 1.09	High

Mean score interpretation: 1.00–2.99: Low, 3.00–500: Moderate, 5.01–7.00: High

Table 2 shows the measurements of construct reliability and convergent validity on the part of the constructs to confirm consistency prior to the modelling of the research factors. The measurements were performed using the confirmatory factor analysis (CFA) tool in the Smart-PLS software. As illustrated, the composite reliability (CR) values attained for the following factors—attitude to fogging technique (0.877), perceived benefits (0.911), perceived risk (0.900), trust in key players (0.877), attitude to nature versus materials (0.895), and religiosity (0.955)—showed that the constructs were consistent. Furthermore, the sum of the average variance extracted (AVE) exceeded a value of 0.5, thereby indicating the reliability of the items in representing the constructs' variances [50].

**Table 2:** Construct consistency and construct reliability

Construct	Item	Loading	CR	AVE	Reliability
Attitude to Fogging Technique	AFT1	0.740	0.877	0.589	YES
	AFT2	0.726			
	AFT3	0.687			
	AFT4	0.788			
	AFT5	0.882			
Perceived Benefit	PB1	0.740	0.911	0.596	YES
	PB2	0.805			
	PB3	0.744			
	PB4	0.75			
	PB5	0.837			
	PB6	0.797			
	PB7	0.709			
Perceived Risk	PR1	0.529	0.900	0.573	YES
	PR2	0.854			
	PR3	0.849			
	PR4	0.905			
	PR5	0.867			
	PR6	0.520			
	PR7	0.663			
Trust in Key Players	TRUST1	0.850	0.877	0.704	YES
	TRUST2	0.861			
	TRUST3	0.804			
Attitude to Nature *versus* Material	NAT1	0.743	0.895	0.634	YES
	NAT2	0.830			
	NAT3	0.894			
	NAT4	0.847			
	NAT5	0.643			
Religiosity	REG1	0.894	0.955	0.729	YES
	REG2	0.848			
	REG3	0.913			
	REG4	0.873			
	REG5	0.898			
	REG6	0.828			
	REG7	0.793			
	REG8	0.771			

Tables 3 and 4 illustrate the assessment of discriminant validity performed using the Fornell-Larcker and Heterotrait-Monotrait Ratio (HTMT) criterion. In the Fornell-Larcker criterion assessment, each variable factor should have a higher square root AVE value than the correlation estimates of the constructs [51, 52]. The study results with regard to the Fornell-Larcker criterion in this study showed a square root AVE value (diagonal elements) that exceeded the total variance shared with the other variable factors (highlighted in bold). The value of HTMT should not exceed 1, in that a value exceeding 1 indicates that the factor is less than the discriminant factual aspect [52, 53]. Next, the assessment of the structural model was performed by evaluating the degree of collinearity in the inner model of the study. Previous studies indicated that the VIF values must be less than 5.00 to make sure no collinearity problem exists [50, 54, 55]. Table 5 shows the results of the collinearity test when the VIF values are less than the threshold of 3.3 [55, 56].

**Table 3:** Fornell-Larcker criterion

	**AFT**	**PB**	**PR**	**TRUST**	**NAT**	**REG**
**AFT**	0.767					
**PB**	0.741	0.772				
**PR**	-0.131	-0.115	0.757			
**TRUST**	0.419	0.398	-0.192	0.839		
**NAT**	0.025	0.060	-0.302	-0.012	0.796	
**REG**	0.173	0.192	-0.048	0.147	-0.189	0.853

Diagonal elements highlighted in bold represents the square root of AVE value that exceeded the total variance shared with other variable factors

**Table 4:** HTMT criterion

	**AFT**	**PB**	**PR**	**TRUST**	**NAT**	**REG**
**AFT**	1					
**PB**	0.855	1				
**PR**	0.170	0.152	1			
**TRUST**	0.509	0.472	0.211	1		
**NAT**	0.114	0.127	0.301	0.104	1	
**REG**	0.186	0.201	0.110	0.199	0.204	1

Discriminant validity aspect is established at the values of HTMT_0.90_

**Table 5:** Collinearity assessment

	PB	PR	AFT
PB	-	-	1.216
PR	1.155	-	1.041
TRUST	1.061	1.022	1.225
NAT	1.153	1.037	-
REG	1.068	1.059	1.046

To answer the research questions related to the proposed hypotheses, the boot-strapping method was performed to identify the path coefficient of the predictors. As shown in Table 6, the t-values indicate the relationship between influencing factors on stakeholders' attitudes towards the fogging technique. The results showed that all the six hypotheses proposed in this study were supported, as the t-values were significant. Specifically, the perceived benefits appeared to be the most important direct predictor of attitude towards fogging (β = 0.678, *p* < 0.01) (Table 6). This finding indicates that stakeholders considered the beneficial aspects of fogging when expressing their positive support for the technique. Trust in key players emerged as the second most important predictor of the stakeholders' attitude towards fogging (β = 0.142, *p* < 0.01) and this predictor also showed a positive association with perceived benefits (β = 0.374, *p* < 0.01) (Table 6). On the other hand, attitude towards nature versus material displayed a strong negative association with perceived risk (β =—0.317, *p* < 0.01), followed by trust in key players (β =—0.183, *p* < 0.01) (Table 6). Stakeholders who were more inclined towards nature as opposed to fulfilling their materialistic needs tended to perceive risk towards fogging. They also perceived more risk with regard to this technique when they had less trust in key players. Interestingly, religiosity was found to have a positive connection with perceived benefit, implying that persons who claimed to be religious were more likely to perceive the benefits of fogging (β = 0.155, *p* < 0.01) (Table 6).

**Table 6:** Path coefficient assessment

	Direct Effects	Standard Error	T-statistic	*P* value	Conclusion
**H1**	0.095	0.060	1.586	0.056	**Not Supported**
**H2**	-0.317	0.042	7.520**	0.000	**Supported**
**H3**	0.374	0.051	7.410**	0.000	**Supported**
**H4**	-0.183	0.049	3.760**	0.000	**Supported**
**H5**	0.142	0.045	3.155**	0.001	**Supported**
**H6**	0.155	0.048	3.213**	0.001	**Supported**
**H7**	-0.086	0.056	1.533	0.063	**Not Supported**
**H8**	-0.001	0.038	0.029	0.318	**Not Supported**
**H9**	0.678	0.034	19.816**	0.000	**Supported**
**H10**	-0.008	0.067	0.124	0.451	**Not Supported**
**H11**	-0.024	0.034	0.708	0.239	**Not Supported**

**p < 0.01, *p < 0.05 (one-tailed)

Table 7 illustrates the analysis of the structural model which comprises the following: i) testing of *R*^*2*^ to test the determination coefficient of the structural model, ii) testing the effect of *f*^2^ size on the impact value of the factors, and iii) testing the relevance of the model predictions with the value of *Q*^*2*^. The value of the determination coefficient (*R*^*2*^) for the attitude towards fogging was 0.568, thus indicating that the perceived benefit and trust in key players accounted for 56.8% of the variance in the attitude towards fogging. On the other hand, the *R*^*2*^ for perceived benefits was 0.186, whereby religiosity and trust in key players accounted for 18.6% of the perceived benefits. The *R*^*2*^ for perceived risk was 0.134, in which trust in key players and attitude towards nature versus material accounted for 13.4% of the perceived risk. The results indicated that the perceived benefits (*f*^2^ = 0.873) had a large effect size on the attitude towards fogging as compared to trust in key players (*f*^2^ = 0.034). This finding demonstrated that the perceived benefit was an important factor, while trust in key players was the second most important factor in explaining and predicting the attitude towards fogging. Next, trust in key players (*f*^2^ = 0.162) and religiosity (*f*^2^ = 0.028) were important predictor factors for the perceived benefits. Lastly, the attitude towards nature versus material (*f*^2^ = 0.112) and trust in key players (*f*^2^ = 0.038) accounted for the effect size on the perceived risk. The predictive relevance (*Q*^*2*^) of the attitude towards fogging was 0.312; the *Q*^*2*^ value of the perceived benefits was 0.103; and the *Q*^*2*^ value of perceived risk was 0.067. A value of *Q*^*2*^ greater than 0 indicates that the exogenous predictor is appropriate to predicting the endogenous predictor.

**Table 7:** Determination of co-efficient (*R*^2^), effect size (*f*^2^) and predictive relevance (*Q*^2^)

	*Determination Coefficient*	*Predictive Relevancy*	*Effect Size f*^*2*^		
	*R*^*2*^	*Q*^*2*^	AFT	PB	PR
AFT	0.568	0.312			
PB	0.186	0.103	0.873 (L)		
PR	0.134	0.067	0.034 (S)		
TRUST				0.162 (M)	0.038 (S)
NAT					0.112 (S)
REG				0.028 (S)	

## Discussion

**Fig. 2:**
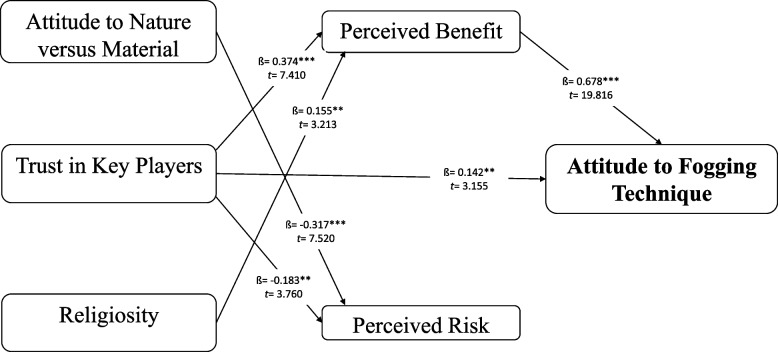
Model for stakeholders’ attitudes toward fogging technique

According to the findings of the study with regard to the PLS model (Fig. 2), when stakeholders recognise a high benefit from the fogging technique, they will display an attitude which supports its implementation. Previous studies have shown that respondents have a good attitude towards any dengue control technique when they perceive its benefits [31, 57]. In addition, a study among the urban community in Titiwangsa, Kuala Lumpur, showed that about 46% of the total 322 respondents in the study believed that chemical was sufficient to control dengue infection [58]. This finding is similar to those of the study by Al-Dubai et al. [10], where 66% of the respondents think fogging is essential for the prevention of dengue infection. Likewise, another study by Sipin et al. [59] also reported that research in South Sulawesi shows fogging is successful if the operation is well-managed, including the use of essential fogging equipment and a proper operator training programme. Meanwhile, Rakhmani et al. [60] reported that community involvement translates into their behaviour being affected by the perception of this fogging technique.

At the same time, the respondents showed moderate concern about the risks associated with fogging. This can be attributed to the use of chemical insecticides as spraying agents. Selvarajoo et al. (2020) reported that about half of the respondents in their survey believed that chemical spraying by health authorities was insufficient for dengue prevention. However, more than 80% want to help reduce the number of dengue cases in their location. Sulistyawati et al. [61] also found that most people in the Yogyakarta neighbourhood of Indonesia need to know the vector control activities of the authorities because they are still determining what they should do during fogging, and they also do not realise that windows and doors should be opened when spraying. In addition, they are concerned about the potential risk of fogging to their health and think that dengue prevention is inappropriate. Ramli et al. [62] also showed that 66.5% of respondents in PPR Batu Muda overlooked opening doors or windows during fogging activities, and unwanted residents still could not obligate health officials for vector control and caused dengue prevention to be less aware. From the findings of the study, it is recommended that health authorities must improve the safety aspect of fogging techniques with safer insecticide options, combined with more environmentally friendly dengue control techniques, as well as convey accurate information to enable stakeholders to see the benefits and have a positive attitude towards the technique associated with the control of dengue.

The results of this study also support the findings by Amin and Hashim [22] and Arham et al. [31], in which trust in key players was the second most important predictor of support for dengue prevention techniques. The findings suggest that Malaysians have positive trust in the key players involved in using or applying the fogging technique to combat the dengue virus. Arham et al. [18, 57, 63] previously noted that respondents placed a high degree of trust in key players in terms of delivering information and performing their jobs competently, thus contributing to the best interest of the community. Toledo et al. [64] suggested that collaborations between the community and key players such as the local government contribute to successful dengue prevention, thus resulting in decreased home risk behaviour, reduced environmental risk, and greater impact on mosquito populations [64].

On the other hand, this study also revealed that the stakeholders' attitudes towards nature versus material had direct negative associations with perceived risk. Specifically, it was shown that the stakeholders in this study were nature-loving people who thought that fogging was not beneficial for the environment, while those who were more materialistic perceived fogging as less risky and more beneficial when it came to controlling dengue. The findings were consistent with the results of Amin et al. [35], who found that this factor was negatively related to perceived risk. Arham et al. [31, 65] highlighted that the respondents were more inclined towards nature than material needs and were more cautious about accepting outdoor residual spraying and Wolbachia-infected Aedes mosquitoes' technique to combat dengue. Therefore, this finding supported those of previous studies which noted that nature-loving people perceived this technique as a risk to nature.

Interestingly, the respondents in this study seemed to be highly committed to their religion. Religiosity has a direct influence on perceived benefits. In a previous study by Arham [66], religiosity has a positive influence on perceived benefits and attitudes toward dengue vaccine. However, in another piece of research relating to stakeholders' points of view regarding genetically modified Aedes mosquitos, it was found that religion was not a predictive factor [18]. According to Mustapa et al. [67] stakeholders in Malaysia who were deeply affiliated to their religion were positively inclined toward new technologies. Therefore, religiosity can be seen as an outcome of informal education practised in the community. The religious practices enabled the individuals to embed appreciation of the environment and the community.

The current study has its limitations, which might also reflect opportunities for future research. For future studies, several variables such as personal values, engagement, or willingness to accept, general concerns, or promises, could be included to determine the attitude towards this technique in order to assess the extent of the community's acceptance of this technique. Additionally, the location coverage of the research can also be extended to other areas throughout Malaysia with a high incidence of dengue cases. Studies could also analyse the effect of ethnicity and religion to obtain a range of opinions or results, since Malaysia is a multi-ethnic country. Furthermore, the prediction of their attitudes to other techniques could also be addressed in the future. Therefore, the responsible parties should attempt to improve the fogging techniques available and develop new environmental-friendly technologies that do not impose any harm on the environment. The results of the study suggest that the group of respondents should be expanded to several stakeholders such as policy makers, non-governmental organizations and industries that are directly and indirectly involved in the control and prevention of dengue, especially in the management of fogging. The location of the study is also not only focused on the Klang Valley, but the study was conducted throughout Malaysia. The findings of this study also suggest that the acceptance of fogging techniques must be focused on the education perspective due to the multi-religion and multicultural backgrounds of the community, given that it plays an important role in shaping their attitudes. Education is a vital factor for consideration as it has the potential to have a direct or indirect effect on the acceptance of fogging techniques. Through proper education, the stakeholders will develop good values, thus appreciating nature and placing their trust in key players to combat dengue effectively. In fact, through education, stakeholders have indicated that fogging is beneficial and relevant when it comes to combatting dengue.

## Conclusion

To conclude, this study confirmed that the factors influencing the stakeholders' attitudes toward fogging in the Klang Valley region should be regarded as a multi-faceted process. Fogging techniques are viewed as beneficial and need to be implemented and continuously improved to combat dengue and ultimately, sustain a healthy society in Malaysia. Although many dengue control techniques have been developed, this study successfully showed that the stakeholders' attitude towards fogging techniques is very positive, and it is still relevant for continued use in the future. To date, this is the first structural equation modelling study conducted in Malaysia to evaluate the attitudes toward fogging using the Smart-PLS approach. These results have important implications for improving the literature related to the factors influencing stakeholders' attitudes towards fogging.

## Supplementary Information

**Additional file 1. **Measurement Items.

## Data Availability

All relevant data are within the manuscript.
