# Supplementary material for: Fogging to combat dengue: factors influencing stakeholders' attitudes in Malaysia
Source: BMC Public Health. 2023 Jun 14;23:1140. doi: 10.1186/s12889-023-16054-3 (PMC10264093; doi:10.1186/s12889-023-16054-3)
Supplement: Supplementary file 1 — Additional file 1. Measurement Items. [file 12889_2023_16054_MOESM1_ESM.docx]

**Additional file 1:** Measurement Items

| **Factors/Items** |
| --- |
| **Attitude to Fogging Technique**  AFT1: Fogging helps to decrease fatality in the community. |
| AFT2: Fogging is necessary. |
| AFT3: Fogging is encouraged. |
| AFT4: Fogging activities should be increased |
| AFT5: The government should provide more financial support to researchers and industries to develop the fogging technique. |
| **Perceived Benefit**  PB1: Fogging will enhance the quality of life. |
| PB2: Fogging is useful to the Malaysian society. |
| PB3: Fogging is useful in preventing dengue fever. |
| PB4: Fogging is effective in eradicating dengue. |
| PB5: Fogging technique is beneficial to me and my family’s health. |
| PB6: The benefits of fogging far out-weigh the risks. |
| PB7: The risks associated with fogging will be addressed by future research studies. |
| **Perceived Risk**  PR1: Level of uncertainties regarding the unknown effects of fogging. |
| PR2: Any harmful effects from fogging will only manifest after a long-term duration. |
| PR3: Fogging will pose threats to future generations. |
| PR4: Fogging may give rise to unknown consequences. |
| PR5: Any danger from fogging may cause a major catastrophe to the Malaysian society |
| PR6: The extent of concern a person has about the potential risks of fogging to their health. |
| PR7: The adverse effects of fogging are harmful. |
| **Trust in Key Players**  TRUST1: Scientists and researchers have achieved a good outcome for the society |
| TRUST2: Pesticides and pharmaceutical industries have achieved a good outcome for the society |
| TRUST3: Government sectors involved in the regulations such as the Ministry of Health and Biosafety Department have achieved a good outcome for the society |
| **Attitude to Nature *versus* Material**  NAT1: A society prefers to preserve nature or use nature to achieve wealth. |
| NAT2: A society with a centrally planned economy or a market-driven economy. |
| NAT3: A society that will stop development at the expense of any risks or accepting any risks in the attainment of wealth. |
| NAT4: A society that optimises the protection of the environment above the economic growth or otherwise |
| NAT5: A society that understands that nature is fragile and can be easily damaged by human actions or can withstand human actions |
| **Religiosity**  REG1: Religion is important in my life. |
| REG2: Religious views are important when I have to make decisions about controversial issues. |
| REG3: Praying is important in my life. |
| REG4: Reading scriptures is important in my life. |
| REG5: Religion is important to answer any questions about the meaning of life. |
| REG6: Religion offers me most is comfort when sorrows and misfortune strike. |
| REG7: I try hard to live all my life according to my religious beliefs. |
| REG8: Nothing can occur without God’s involvement in the process. |
